# Trends of Industry Payments in Neurology Subspecialties

**DOI:** 10.7759/cureus.9492

**Published:** 2020-07-31

**Authors:** Krishna Nalleballe, Karthika Durga Veerapaneni, Yohei Harada, Poornachand Veerapaneni, Narenraj Arulprakash, Jose R Lopez-Castellanos, Sen Sheng, Julia Rowen, Vasuki Dandu, Sanjeeva Onteddu, Suman Siddamreddy

**Affiliations:** 1 Neurology/Stroke, University of Arkansas for Medical Sciences (UAMS), Little Rock, USA; 2 Neurology, University of Arkansas for Medical Sciences (UAMS), Little Rock, USA; 3 Pharmacy, University of Arkansas for Medical Sciences (UAMS), Little Rock, USA; 4 Neurology, Baptist Health Medical Center, Little Rock, USA; 5 Internal Medicine, Baptist Health Medical Center, Little Rock, USA

**Keywords:** health economics, industry payment, open payment program, disparities, neurologist

## Abstract

Background

Open Payments is a national disclosure program to promote transparency by the public disclosure of financial relationships between the pharmaceutical and medical device industries and physicians.

Objective

To explore payments from the industry to physicians in various neurology subspecialties.

Methods

Open Payments Program (OPP) data (https://openpaymentsdata.cms.gov) on industry-to-physician payments for the years 2014-2018 were extracted for general neurology, neuromuscular, neurophysiology, and vascular neurology. The data were then analyzed to explore trends in payments for various subspecialties and to identify the possible factors underlying these trends.

Results

Overall, industry-to-physician payments for neurology subspecialties increased by 16% from 2014 to 2018. The introduction of newer drugs in a subspecialty was likely the driving factor for higher industry payments. Nearly half of the total industry-to-physician payments were for the subspecialty of multiple sclerosis (MS)/Neuroimmunology; this coincided with Aubagio and Copaxone being the top two medications associated with the highest industry payments in 2014, Aubagio, and Lemtrada in 2018. A significant increase in spending percentages for headache, neuromuscular disorders, and movement disorders was observed while a relative decrease in the payments for MS/neuroimmunology and epilepsy was identified; these trends coincide with the introduction of new drugs such as Aimovig, Neuplazid, Nusinersen, and Austedo for headache, neuromuscular and movement disorders.

Conclusions

From 2014 to 2018, the total industry-to-physician payments for neurology subspecialties increased while the distribution of industry-to-physician payments for various neurology subspecialties showed notable changes. The introduction of newer medications in a subspecialty coincided with higher industry payments. Identification of these trends and potential motives of the industry spending is critical to address any potential physician bias in prescribing medications.

## Introduction

Financial transactions between the industry and physicians introduce potential conflicts of interest [1-2], which may translate to patient care [3]. To improve transparency, the Sunshine Act, implemented in 2010, mandated the disclosure of industry payments to physicians [4]. Industry payment trends have since been studied for several specialties [5-10]; however, the literature on trends of payments for neurological subspecialties is lacking [11]. We aimed to explore payments from pharmaceutical and device manufacturing companies to various subspecialties of neurology.

## Materials and methods

The Open Payments Program (OPP) data (https://openpaymentsdata.cms.gov) on industry-to-physician payments for the years 2014-2018 were extracted for general neurology, neuromuscular, neurophysiology, and vascular neurology. Subspecialty payments data for 2014-2018 were combined into a single dataset and the variable ‘name of associated covered drug or biological’ was used to sort the combined dataset. Type of ‘drug or biological’ for each data point was then studied to ascertain the subspecialty; for example, Tysabri or Tecfidera would suggest the ‘multiple sclerosis/neuroimmunology’ subspecialty while Vimpat or Aptiom would belong to the ‘epilepsy’ subspecialty. In the ‘associated covered drug or biological’ field, we excluded missing data (comprising approximately 3%) and drugs or biologicals with a frequency of fewer than 50 times (around 1%). All payment categories were analyzed, including food, travel, research, education, and consulting fees. All data analysis was conducted in SAS version 9.4 (SAS Institute, Cary, NC).

## Results

In 2014, industry-to-physician payments for all neurology subspecialties in the category of drugs and devices were 64 million USD. The three neurology subspecialties receiving the most industry payments were: multiple sclerosis/neuroimmunology (57.1% of total payments), movement disorders (14.7%), and epilepsy (14.3%) (Table 1).

**Table 1:** Industry payments by specialty in 2014 and 2018 MS - Multiple sclerosis; USD - United States dollars

Specialty	2014 (million USD (%))	2018 (million USD (%))
MS/Neuroimmunology	36.5 (57.1%)	35.1 (46.8%)
Movement	9.6 (14.7%)	13.6 (18.1%)
Epilepsy	9.0 (14.3%)	6.8 (9.0%)
Headache	6.6 (10.2%)	14.7 (19.6%)
Stroke	2.1 (3.4%)	2.3 (3.1%)
Neuromuscular	1.3 (0.2%)	2.5 (3.4%)
Total	64	75

Of the top 10 medications, seven drugs were for multiple sclerosis, and one each was for movement disorders, headache, and epilepsy (Table 2).

**Table 2:** Top 10 drugs: 2014 and 2018 MS - Multiple sclerosis

2014	Specialty	Drug	2018	Specialty	Drug
1	MS/Neuroimmunology	Aubagio	1	MS/Neuroimmunology	Aubagio
2	MS/Neuroimmunology	Copaxone	2	MS/Neuroimmunology	Lemtrada
3	Movement Disorders	Azilect	3	Headache and Pain	Aimovig
4	MS/Neuroimmunology	Tecfidera	4	MS/Neuroimmunology	Tysabri
5	Headache and Pain	Botox	5	MS/Neuroimmunology	Ocrevus
6	MS/Neuroimmunology	Tysabri	6	MS/Neuroimmunology	Tecfidera
7	MS/Neuroimmunology	Plegridy	7	Movement Disorders	Nuplazid
8	MS/Neuroimmunology	Gilenya	8	Movement Disorders	Austedo
9	MS/Neuroimmunology	Ampyra	9	Headache and Pain	Ajovy
10	Epilepsy	Aptiom	10	Epilepsy	Aptiom

By 2018, the industry-to-physician payments increased by 16% to a total of 75 million USD. The three subspecialties receiving the most industry payments in 2018 were: multiple sclerosis/neuroimmunology (46.8% of total payments), headache (19.6%), and movement disorders (18.1%). Of the top 10 medications, five drugs were for multiple sclerosis, two each for headache and movement disorders, and one was for epilepsy (Table 2). From 2014 to 2018, there were notable changes in the subspecialty distribution of these industry-to-physician payments (Table 1). For example, payments increased for medications related to headache (from 10.2% in 2014 to 19.6% in 2018), neuromuscular disorders (from 0.2 to 3.4%), and movement disorders (14.7% to 18.1%), while the payments decreased for medications related to multiple sclerosis/neuroimmunology (from 57.1% to 46.8%) and epilepsy (from 14.3% to 9%) and remained stable for stroke-related medications (3.4% to 3.1%).

## Discussion

In this study, we used publicly available databases to explore and report payments from industry to various neurology subspecialties between 2014 and 2018.

There was a significant increase in the total payment to neurology from 2014 to 2018 by 16%. Among them, industry payments for movement disorders, headache, and neuromuscular were increased. Especially, headache and neuromuscular had a substantial increase in these four years. It is consistent with the fact that headache drugs took the third and ninth place of the highest-paid drugs in 2018. There had been no headache medications approved by the Food and Drug Administration (FDA) from 2014 until 2018 when two new drugs (Fremanezumab and Erenumab) came to market (https://www.fda.gov/drugs/new-drugs-fda-cders-new-molecular-entities-and-new-therapeutic-biological-products/novel-drug-approvals-2018) (Table 3).

**Table 3:** FDA approved drugs 2014-2018 MS - Multiple sclerosis; FDA - Food and Drug Administration

Year	Drug Name	Brand name	Indication
2014	Droxidopa	Northera	Neuromuscular
	Florbetaben	Neuraceq	Dementia
	Peginterferon beta 1a	Plegridy	MS/Neuroimmunology
2015	Idarucizumab	Praxbind	Stroke
2016	Brivaracetam	Briviact	Epilepsy
	Pimavanserin	Nuplazid	Movement
	Daclizumab	Zinbryta	MS/Neuroimmunology
	Eteplirsen	Exondys 51	Neuromuscular
	Nusinersen	Spinraza	Neuromuscular
2017	Edaravone	Radicava	Neuromuscular
	Valbenazine	Ingrezza	Movement
	Deutetrabenazine	Austedo	Movement
	Ocrelizumab	Ocrevus	MS
	Safinamide	Xadago	Movement
	Deflazacort	Emflaza	Neuromuscular
2018	Amifampridine	Firdapse	Neuromuscular
	Inotersen	Tegsedi	Neuromuscular
	Fremanezumab	Ajovy	Headache
	Stiripentol	Diacomit	Epilepsy
	Migalastat	Galafold	Neuromuscular
	Patisiran	Onpattro	Neuromuscular
	Cannabidiol	Epidioloex	Epilepsy
	Erenumab	Aimovig	Headache

Similarly, there have been many breakthrough advances in the neuromuscular disease field such as Nusinersen for spinal muscular atrophy. According to the FDA data, among 23 newly approved drugs with a neurological indication between 2014 and 2018, nine of them were indicated for neuromuscular disorders. It is also reported that there are currently nearly 200 products in the therapeutic pipeline for neuromuscular disorders and we presume the growth of this field will continue. On the other hand, there is a slight reduction in MS/neuroimmunology, and epilepsy in four years, although six out of the 10 highest paid drug in 2018 were therapies for MS/neuroimmunology. This could be related to the fact that there were no newly FDA-approved MS/neuroimmunology drugs and only one epilepsy drug (Cannabidiol) in 2018. Given that the industry payment for each drug includes the fee for food, travel, research, education, and consulting, the newly approved drug would likely to be received more investment to increase awareness among neurologists. Further studies are needed to evaluate if there is any potential for influence on thought leaders in the field, as has been published before, along with the focus on educational components for newly approved medications [12-14].

## Conclusions

From 2014 through 2018, the distribution of industry-to-physician payments for various neurology subspecialties showed notable changes. Payments to the subspecialties of headache, neuromuscular disorders, and movement disorders increased, likely related to the introduction of newer medications in these fields. Physician education and knowledge of the trends and potential motives of industry spending is critical to address any potential bias in prescribing medications when alternatives may be available.
